# IL-1ra Secreted by ATP-Induced P2Y_2_ Negatively Regulates MUC5AC Overproduction via PLC*β*3 during Airway Inflammation

**DOI:** 10.1155/2016/7984853

**Published:** 2016-02-29

**Authors:** Jee-Yeong Jeong, Jiwook Kim, Bokyoum Kim, Joowon Kim, Yusom Shin, Judeok Kim, Siejeong Ryu, Yu-Mi Yang, Kyoung Seob Song

**Affiliations:** ^1^Department of Biochemistry, Kosin University College of Medicine, Busan 49267, Republic of Korea; ^2^Cancer Research Institute, Kosin University College of Medicine, Busan 49267, Republic of Korea; ^3^Department of Anesthesiology and Pain Medicine, Kosin University College of Medicine, Busan 49267, Republic of Korea; ^4^Department of Oral Biology, BK21 PLUS Project, Yonsei University College of Dentistry, Seoul 03722, Republic of Korea; ^5^Department of Physiology, Kosin University College of Medicine, Busan 49267, Republic of Korea; ^6^Institute of Medicine, Kosin University College of Medicine, Busan 49267, Republic of Korea

## Abstract

Mucus secretion is often uncontrolled in many airway inflammatory diseases of humans. Identifying the regulatory pathway(s) of mucus gene expression, mucus overproduction, and hypersecretion is important to alleviate airway inflammation in these diseases. However, the regulatory signaling pathway controlling mucus overproduction has not been fully identified yet. In this study, we report that the ATP/P2Y_2_ complex secretes many cytokines and chemokines to regulate airway inflammation, among which IL-1 receptor antagonist (IL-1ra) downregulates* MUC5AC* gene expression via the inhibition of G*α*q-induced Ca^2+^ signaling. IL-1ra inhibited IL-1*α* protein expression and secretion, and vice versa. Interestingly, ATP/P2Y_2_-induced IL-1ra and IL-1*α* secretion were both mediated by PLC*β*3. A dominant-negative mutation in the PDZ-binding domain of PLC*β*3 inhibited ATP/P2Y_2_-induced IL-1ra and IL-1*α* secretion. IL-1*α* in the presence of the ATP/P2Y_2_ complex activated the ERK1/2 pathway in a greater degree and for a longer duration than the ATP/P2Y_2_ complex itself, which was dramatically inhibited by IL-1ra. These findings suggest that secreted IL-1ra exhibits a regulatory effect on ATP/P2Y_2_-induced* MUC5AC* gene expression, through inhibition of IL-1*α* secretion, to maintain the mucus homeostasis in the airway. Therefore, IL-1ra could be an excellent modality for regulating inflamed airway microenvironments in respiratory diseases.

## 1. Introduction

Mucus overexpression and hypersecretion in the human respiratory track are a critical characteristic of a number of pulmonary diseases including rhinitis, sinusitis, asthma, COPD, and cystic fibrosis [1]. A family of highly glycosylated proteins, named mucins, has been identified as important constituents of mucus. However, the physiological functions of mucins have not been fully understood yet. In the human body, several innate immune systems downregulate mucin overexpression and hypersecretion, thus decrease inflamed microenvironments, and maintain homeostasis. Identification of the regulatory mechanisms which lead to control mucin overexpression and hypersecretion in pulmonary diseases is essential for developing new therapeutic drugs. Since MUC5AC (for human; Muc5ac for nonhumans) is one of the major mucins in human airway diseases, the mechanisms regulating* MUC5AC* gene expression have been considered critical to identify strategies to prevent airway mucus overproduction.

The IL-1 family of molecules consists of two agonists, IL-1*α* and IL-1*β*, and a specific receptor antagonist called IL-1ra, which interact with two different receptors, IL-1R type I (IL-1RI) and IL-1R type II (IL-1RII) [2, 3]. The IL-1 family plays an important role in maintaining homeostasis against microorganisms, air pollutants, exotoxins, and airborne particulate matters in the human respiratory track. It also takes an important part in the innate immune system, which further regulates functions of the adaptive immune system [4]. The balance between IL-1 and IL-1ra in the human lung may affect the development of inflammatory diseases. Voelkel et al. reported that treatment with IL-1ra reduced pulmonary hypertension generated by monocrotaline in rats [5], but IL-1*β*, one of the earliest mediators secreted during a proinflammatory response, is present at high levels in the lungs of patients with chronic lung diseases [6]. However, other secreted inflammatory mediators can also regulate their intracellular expressions, thereby further modulating cytokine-regulated MUC5AC overproduction.

The purinergic P2Y_2_ receptor (P2Y_2_R) is highly expressed in the apical membrane of the respiratory track [7–9]. Extracellular nucleotides bound to the P2Y_2_R have been shown to induce a number of physiological phenomena in a variety of human cell types and tissues [10]. Recently, P2Y_2_R deficiency was shown to attenuate IFN*γ*, IL-17, and TNF*α* secretion in mice [11]. P2Y couples to the G-protein subtype, G*α*q, and the G*α*q protein regulates Ca^2+^ influx upon stimulation with ligands [12]. Next, elevated [Ca^2+^]_i_ increases PLC*β*3. PLC*β* isotypes have a long C-terminal region (~400 residues) with relatively low homology among family members [13]. This C-terminal region (residues 903–1142 of PLC*β*1) is required for binding and stimulation by G*α*q [14]. Moreover, PLC*β* isotypes possess short consensus sequences known as postsynaptic density- (PSD-) 95/disc large/ZO-1- (PDZ-) binding motifs. The PDZ-binding motif is a well-known short linear motif that interacts with other PDZ proteins [15]. The regulatory mechanism of PLC*β*3-mediated cytokine secretion to control* MUC5AC* gene expression and the signal molecules involved, especially those involved in downstream signaling of PLC*β*3, has not yet been demonstrated in the human airway epithelium.

Here, we report that ATP induces IL-1ra overexpression and secretion via the P2Y_2_ receptor in human airway epithelial cells. IL-1ra secretion was associated with the P2Y_2_-G*α*q-[Ca^2+^]_i_-PLC*β*3-ERK1/2 pathway, sequentially. Secreted IL-1ra inhibits ATP/P2Y_2_-induced* MUC5AC* gene expression, but IL-1*α* significantly increases the expression of* MUC5AC*. The results of the present study reveal a novel mechanism for intra- and intercellular signaling pathways that control airway inflammation, thus, providing an opportunity for the development of novel therapeutic modalities to treat respiratory diseases.

## 2. Materials and Methods

### 2.1. Materials

ATP and Apyrase were purchased from Sigma Aldrich (St. Louis, MO). The IL-1ra and IL-1*α* ELISA kits were purchased form R&D Systems (Minneapolis, MN). siRNA targeting P2Y_2_ was synthesized by Bioneer (Daejeon, Korea): P2Y_2_, GAGGAAGGUGGCUUACCAA (dTdT); and negative control CCUACGCCACCAAUUUCGU (dTdT). All PLC*β*3 isotypes were kindly provided by Dr. Pann-Ghill Suh (School of Life Sciences, Ulsan National Institute of Science and Technology, Ulsan, Korea).

### 2.2. Cytokine Assay

The cytokine assay was performed using a Human Cytokine Array Panel A kit (R&D Systems) according to the manufacturer's instructions. Briefly, cells were plated in 6-well plates one day prior to transfection with either P2Y_2_ or siRNA-P2Y_2_ using FuGENE 6 (Roche; Indianapolis, IN). Twenty-four hours after transfection, serum-starved cells were treated with ATP for 4 hours. After treatment, the supernatant was assayed according to the kit instructions.

### 2.3. RT-PCR

Real-time PCR was performed using a BioRad iQ iCycler Detection System (BioRad Laboratories; Hercules, CA) with iQ SYBR Green Supermix. Reactions were performed in a total volume of 20 *μ*L including 10 *μ*L 2x SYBR Green PCR Master Mix, 300 nM of each primer, and 1 *μ*L of previously reverse-transcribed cDNA template. The following primers were used: MUC5AC, forward 5′-CAGCCACGTCCCCTTCAATA-3′ and reverse 5′-ACCGCATTTGGGCATCC-3′; *β*_2_-microglobulin used as a reference for normalization, forward 5′-CGCTCCGTGGCCTTAGC-3′ and reverse 5′-GAGTACGCTGGATAGCCTCCA-3′. Parameters were 95°C for 10 m, followed by 40 cycles of 95°C for 15 s, 60°C for 30 s, and 72°C for 30 s. All reactions were performed in triplicate. Relative quantity of MUC5AC mRNA was obtained using a comparative cycle threshold method and was normalized using *β*_2_-microglobulin as an endogenous control.

### 2.4. Western Blot Analysis

NCI-H292 cells were grown to confluence in 6-well plates. After treatment with ATP, cells were lysed with 2x lysis buffer (250 mM Tris-Cl (pH 6.5), 2% SDS, 4%  *β*-mercaptoethanol, 0.02% bromphenol blue, and 10% glycerol). Equal amounts of whole cell lysates were resolved by 10–15% SDS-PAGE and transferred to polyvinylidene difluoride membranes (Millipore, Bedford, MA). Membranes were blocked with 5% skim milk in Tris-buffered saline (50 mM Tris-Cl (pH 7.5), 150 mM NaCl) for 2 h at room temperature. Blots were then incubated overnight with primary antibodies in TTBS (0.5% Tween 20 in Tris-buffered saline). After washing with TTBS, blots were further incubated for 45 min at room temperature with anti-rabbit or anti-mouse antibody (Cell Signaling; Danvers, MA) in TTBS and visualized using the ECL system (GE Healthcare; Uppsala, Sweden).

### 2.5. Measurement of Intracellular Ca^2+^ Concentration ([Ca^2+^]_i_)

NCI-H292 cells were seeded on cover glasses in 35-mm dishes (5 × 10^4^ cells). After 24 h, cells were transfected with P2Y_2_-expressing construct. The cells expressing P2Y_2_ in physiological salt solution [140 mM NaCl, 5 mM KCl, 1 mM MgCl_2_, 1 mM CaCl_2_, 10 mM HEPES, 10 mM glucose, and 310 mOsm, (pH 7.4)] were incubated with 5 *μ*M Fura-2/AM (TEFLabs Inc., Austin, TX) and 0.05% pluronic F-127 for 60 min at room temperature. Fura-2 fluorescence was measured at the appropriate excitation wavelengths (340/380 nm), and emission at 510 nm wavelengths (ratio = F340/F380) using a Molecular Devices (Downingtown, PA) imaging system. The emitted fluorescence was monitored with a charge-coupled device camera (Photometrics, Tucson, AZ) attached to an inverted microscope. Fluorescence images were obtained at 2 sec intervals. All data were analyzed using MetaFlour software (Molecular Devices).

### 2.6. Statistical Analysis

Data are presented as the mean ± S.D. of at least three independent experiments. Where appropriate, statistical differences were assessed by the Wilcoxon Mann-Whitney test. *p* values less than 0.05 were considered statistically significant.

## 3. Results

### 3.1. ATP Secretes IL-1ra Extracellularly via the G*α*q-Coupled P2Y_2_ Receptor in NCI-H292 Cells

**Figure 1:**
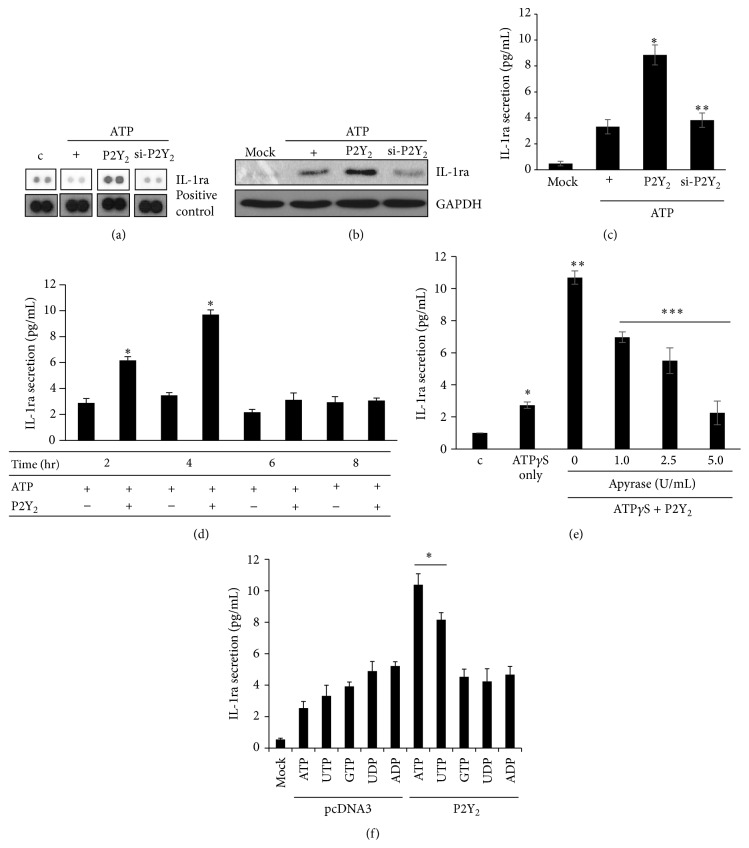
ATP induces IL-1ra secretion and overexpression through a P2Y_2_-dependent pathway in NCI-H292 cells. (a) A construct expressing wild-type P2Y_2_ or siRNA-P2Y_2_ was transiently transfected into NCI-H292 cells. The cells were washed and serum-starved overnight. They were subsequently treated with ATP for 4 hours and a cytokine assay was performed. (b) Cells were transfected with either a construct driving the expression of wild-type P2Y_2_ or P2Y_2_-specific siRNA. Cells were then treated with ATP for four hours and their supernatants collected. The total cell lysates were analyzed by Western blot analysis. Mock transfection was performed with parent* pcDNA3* only. (c) The levels of IL-1ra in the cell supernatants were measured by ELISA. ^*∗*^ *p* < 0.05 compared with ATP only; ^*∗∗*^ *p* < 0.05 compared with ATP/P2Y_2_-treated cells. (d) Cells were transfected with* pcDNA3.1::P2Y*_*2*_ or* pcDNA3.1* and were treated with ATP (10 *μ*M) for the indicated times. The concentrations of IL-1ra in the resultant supernatants were measured by ELISA. Values shown are the means ± SD of three technical replicates from a single experiment. ^*∗*^ *p* < 0.05 compared with the ATP only treatment each time. (e) After transfection with wild-type P2Y_2_, the cells were subsequently treated with Apyrase for 4 hours prior to treatment with ATP*γ*S (10 *μ*M) for 4 hours. IL-1ra secretion was measured by ELISA. Values shown represent the means ± SDs of three technical replicates from a single experiment. ^*∗*^ *p* < 0.05 compared to the control, ^*∗∗*^ *p* < 0.05 compared to ATP only, and ^*∗∗∗*^ *p* < 0.05 compared to ATP/P2Y_2_-treated cells. (f) Cells transfected with either pcDNA3 or wild-type P2Y_2_ were treated with nucleotides (10 *μ*M each) for 4 hours. IL-1ra secretion was measured by ELISA. Values shown represent the means ± SDs of three technical replicates from a single experiment. ^*∗*^ *p* < 0.05 compared with control. Data shown are representative of three independent experiments.

In our previous study [12], ATP was shown to induce* MUC5AC* gene expression via the P2Y_2_ receptor and the MAPK pathway in NCI-H292 (mucoepidermoid pulmonary carcinoma) cells. To determine whether ATP treatment induces or inhibits secretion of cytokines that may regulate the respiratory microenvironment to maintain homeostasis, we performed the cytokine array with cell culture medium after treatment of NCI-H292 cells with ATP for 4 hours (Figure 1(a)). The secretion of IL-1 receptor antagonist (IL-1ra) was increased in cells overexpressing P2Y_2_, but not in cells transfected with P2Y_2_-specific siRNA (Figure 1(a)). To examine whether P2Y_2_ is essential for IL-1ra secretion and overproduction, we performed Western blot analysis (Figure 1(b)) and IL-1ra-specific ELISA (Figure 1(c)) in the presence or absence of transfection with the constructs for wild-type P2Y_2_ or siRNA-P2Y_2_ expression. Treatment with ATP alone for four hours did not induce either IL-1ra secretion or overproduction; however, ATP did induce IL-1ra secretion and overproduction in cells overexpressing P2Y_2_. Conversely, siRNA-mediated knockdown of P2Y_2_ dramatically decreased ATP-mediated induction of IL-1ra secretion and overproduction. After showing that IL-1ra secretion was accelerated early by the inflammatory response, we examined whether ATP/P2Y_2_ signaling could induce IL-1ra overproduction. We previously showed that IL-1*α* secretion and overproduction were upregulated by the ATP/P2Y_2_ signaling complex in airway epithelium [16]. Extracellular secretion of IL-1ra reached its maximum level after 4 hours of treatment with ATP/P2Y_2_ and decreased thereafter (Figure 1(d)). Next, to investigate whether extracellular ATP secreted by exogenous ATP-induced P2Y_2_ activation is required for this phenomenon, cells were incubated with Apyrase, an ATPase/ADPase, in order to block the action of secreted ATP, followed by incubation with ATP*γ*S, which is not hydrolyzed by Apyrase. IL-1ra secretion induced by ATP*γ*S treatment was decreased in an Apyrase dose-dependent manner (Figure 1(e)), indicating that the secreted ATP rather than the treated ATP is critical for increased IL1-ra secretion. In addition, we also compared the stimulatory activities of various nucleotides for IL-1ra secretion. Only ATP or UTP could dramatically increase the secretion of IL-1ra in cells expressing P2Y_2_ (Figure 1(f)). The effects of nucleotides on IL-1ra secretion were as follows: ATP > UTP > GTP = ADP = UDP. These results suggest that intracellular secretion of IL-1ra is stimulated by the ATP/P2Y_2_ complex, which plays an important immunological role to control inflammatory signaling in airway cells. These results demonstrated that cells activated by the ATP/P2Y_2_ complex could promote secretion of ATP, which may trigger signal transduction pathways in either an autocrine or paracrine manner, resulting in subsequent secretion of IL-1ra from the cells. ATP/P2Y_2_ is appeared to be essential for extracellular secretion and overproduction of IL-1ra in airway epithelial cells.

### 3.2. IL-1ra Antagonizes IL-1*α* Secretion and Overproduction to Inhibit IL-1*α*-Induced* MUC5AC* Gene Expression

**Figure 2:**
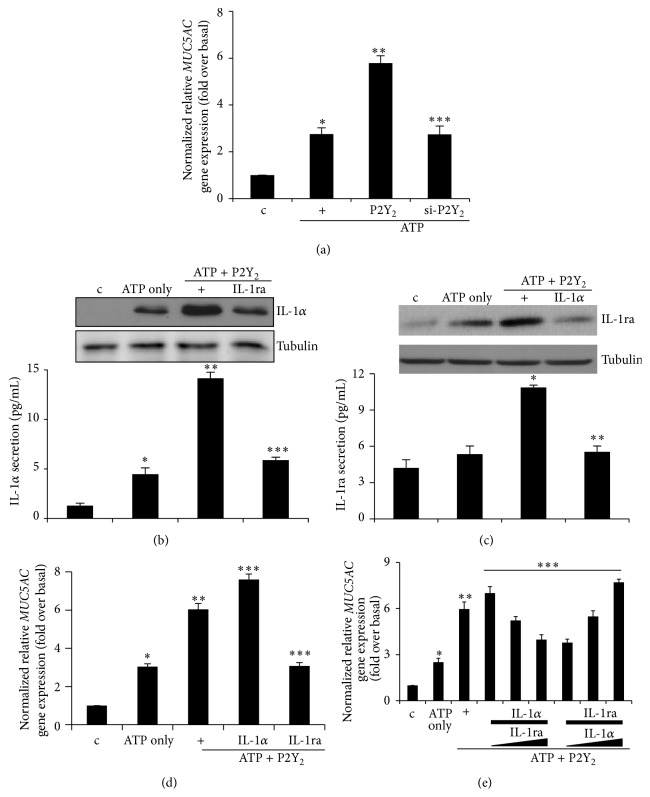
IL-1ra inhibits IL-1*α*-induced* MUC5AC* gene expression via inhibiting IL-1*α* secretion and overproduction. (a) Cells were transfected with either a construct driving the expression of wild-type P2Y_2_ or siRNA specific for P2Y_2_. Cells were then treated with ATP for 24 hours prior to the generation of total cell lysates, and then qRT-PCR for* MUC5AC* transcript was performed. ^*∗*^ *p* < 0.05 compared to the control, ^*∗∗*^ *p* < 0.05 compared to ATP only, and ^*∗∗∗*^ *p* < 0.05 compared to ATP/P2Y_2_-treated cells. (b) After transfection with wild-type P2Y_2_ or siRNA-P2Y_2_, the cells were subsequently treated with IL-1ra (50 ng/mL) for 4 hours prior to treatment with ATP for 4 hours. IL-1*α* production/secretion was measured by Western blot and ELISA, respectively, using the identical set of samples. Values shown represent the means ± SDs of three technical replicates from a single experiment. Two additional experiments showed similar results. ^*∗*^ *p* < 0.05 compared to the control, ^*∗∗*^ *p* < 0.05 compared to ATP only, and ^*∗∗∗*^ *p* < 0.05 compared to ATP/P2Y_2_-treated cells. (c) Cells were transfected with either a construct driving the expression of wild-type P2Y_2_ or siRNA specific for P2Y_2_. The cells were subsequently treated with IL-1*α* (50 ng/mL) for 4 hours prior to treatment with ATP for 4 hours. IL-1ra production/secretion was measured by Western blot and ELISA. Values shown represent the means ± SDs of three technical replicates from a single experiment. ^*∗*^ *p* < 0.05 compared to ATP only and ^*∗∗*^ *p* < 0.05 compared to ATP/P2Y_2_-treated cells. (d) After transfection with wild-type P2Y_2_, the cells were subsequently treated with either IL-1ra (50 ng/mL) or IL-1*α* (50 ng/mL) for 4 hours prior to treatment with ATP for 24 hours. qRT-PCR for* MUC5AC* transcript was performed. ^*∗*^ *p* < 0.05 compared to the control, ^*∗∗*^ *p* < 0.05 compared to ATP only, and ^*∗∗∗*^ *p* < 0.05 compared to ATP/P2Y_2_-treated cells. (e) After transfection with wild-type P2Y_2_, the cells were treated with both IL-1*α* (50 ng/mL) and IL-1ra (0, 100, 150 ng/mL) or both IL-1ra (50 ng/mL) and IL-1*α* (0, 100, 150 ng/mL) for 4 hours prior to treatment with ATP for 24 hours. qRT-PCR for* MUC5AC* transcript was performed. ^*∗*^ *p* < 0.05 compared to the control, ^*∗∗*^ *p* < 0.05 compared to ATP only, and ^*∗∗∗*^ *p* < 0.05 compared to ATP/P2Y_2_-treated cells. Data shown are representative of three independent experiments.

We previously showed that* MUC5AC* gene expression was upregulated by LPS-induced secretion of ATP [17]. In addition, IL-1*α* secretion was also mediated by ATP/P2Y_2_ signaling to induce* MUC8* gene expression [16]. Thus, we hypothesized that IL-1ra secreted by ATP/P2Y_2_ might regulate IL-1*α* secretion and overproduction to antagonize IL-1*α* function. Moreover, we speculated that IL-1*α* secretion could alter IL-1ra secretion and overproduction, reciprocally. First, we confirmed that the P2Y_2_ complex was essential for ATP-induced* MUC5AC* gene expression in human airway epithelial NCI-H292 cells (Figure 2(a)) [12]. Second, P2Y_2_-transfected cells were treated with either ATP only or ATP and IL-1ra (50 ng/mL) for 4 hours. We found that IL-1ra treatment inhibited ATP/P2Y_2_-induced IL-1*α* overproduction by Western blot analysis (Figure 2(b), upper panel) and its secretion by ELISA (Figure 2(b), lower panel). In the same way, IL-1*α* treatment also inhibited IL-1ra secretion and overproduction, reciprocally (Figure 2(c)). Next, we investigated whether secreted IL-1*α* or IL-1ra by ATP/P2Y_2_ could increase* MUC5AC* gene expression in airway epithelial cells. Interestingly, whereas IL-1ra treatment dramatically decreased ATP/P2Y_2_-mediated induction of* MUC5AC* gene expression, IL-1*α* treatment slightly increased expression of* MUC5AC* (Figure 2(d)). To examine the role of IL-1ra in IL-1*α* secretion by cells expressing P2Y_2_, we cotreated cells with both IL-1*α* (50 ng/mL) and IL-1ra in a dose-dependent manner (0, 100, and 150 ng/mL) as a competitive inhibitor, reciprocally. Because IL-1ra has the same binding strength for the IL-1 receptor as IL-1*α*, the intrinsic agonist activity of IL-1*α* may abolish its own function. High dosages (100 and 150 ng/mL) of IL-1ra markedly reduced IL-1*α* (50 ng/mL)-induced* MUC5AC* gene expression; however, IL-1*α* dosages (100 and 150 ng/mL) dramatically increased IL-1ra (50 ng/mL)-reduced expression (Figure 2(e)). These results indicate that IL-1ra secretion antagonizes physiological phenomena induced by IL-1*α*. Therefore, IL-1ra secreted by the ATP/P2Y_2_ complex can regulate airway inflammation to decrease* MUC5AC* gene expression in airway epithelial cells.

### 3.3. IL-1ra Abolishes G*α*q-Mediated PLC*β*3 Activation and Ca^2+^ Secretion

**Figure 3:**
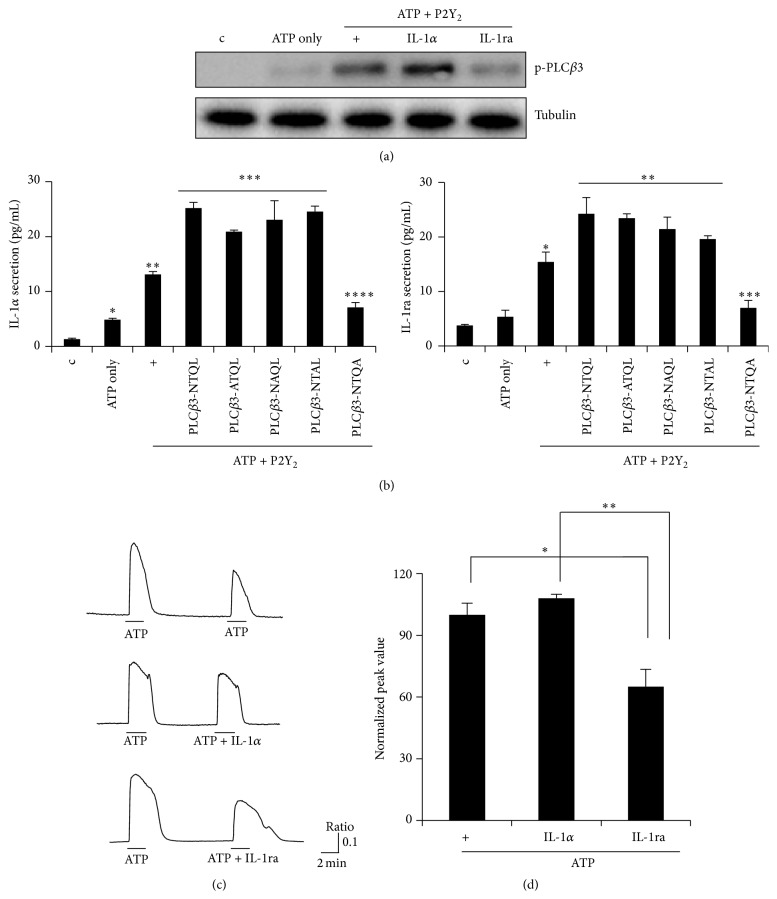
Effect of PLC*β*3 activation on IL-1ra and IL-1*α* secretion. (a) After transfection with wild-type P2Y_2_, the cells were subsequently treated with IL-1ra or IL-1*α* for 4 hours prior to treatment with ATP for 5 min. PLC*β*3 activation was measured by Western blot. Tubulin was used as a loading control. (b) Cells were transiently transfected with both wild-type P2Y_2_ and wild-type (^1231^NTQL^1234^), dominant-negative PLC*β*3 ATQL (N1231A), NAQL (T1232A), NTAL (Q1233A), or NTQA (L1234A) construct. Each of the individual residues of the PDZ-binding motif (NTQL) of PLC*β*3 was mutated to Ala, respectively. Cells were serum-starved overnight and then treated with ATP for 4 hours, after which supernatants were harvested for ELISA. For IL-1*α* ELISA (left panel), ^*∗*^ *p* < 0.05 compared to the control, ^*∗∗*^ *p* < 0.05 compared to ATP only, ^*∗∗∗*^ *p* < 0.05 compared to ATP/P2Y_2_-treated cells, and ^*∗∗∗∗*^ *p* < 0.05 compared to ATP/P2Y_2_/wild-type PLC*β*3-treated cells. For IL-1ra ELISA (right panel), ^*∗*^ *p* < 0.05 compared to ATP only, ^*∗∗*^ *p* < 0.05 compared to ATP/P2Y_2_-treated cells, and ^*∗∗∗*^ *p* < 0.05 compared to ATP/P2Y_2_/wild-type PLC*β*3-treated cells. Data shown are representative of three independent experiments. (c) After transfection with wild-type P2Y_2_, the cells were treated with ATP, IL-1*α*, or IL-1ra. Ca^2+^ release was measured by adding increasing concentrations of Fura-2. See in Section 2. (d) Normalized peak value of the results in Figure 4(a). ^*∗*^ *p* < 0.05 compared to ATP-treated cells, ^*∗∗*^ *p* < 0.05 compared to ATP/P2Y_2_/IL-1*α*-treated cells.

To determine whether IL-1ra or IL-1*α* can affect phospholipase C (PLC) *β*3 activation, we performed Western blot analysis with a phospho-specific PLC*β*3 antibody (Figure 3(a)). IL-1ra dramatically inhibited PLC*β*3 phosphorylation, whereas IL-1*α* slightly increased phosphorylation of PLC*β*3. PLC*β*3 has short consensus sequences known as postsynaptic density-95/discs large/ZO-1- (PDZ-) binding motifs that consist of the amino acids X(S/T)X(V/L)-COOH at the immediate C-terminus [12, 15]. The PDZ domain of PLC*β*3 is critical for binding to the four amino acids at the C-terminus of target proteins [18–20]. To investigate whether the PDZ domain of the PLC*β*3 C-terminus plays a role in ATP/P2Y_2_-activated IL-1ra and IL-1*α* secretion and overproduction, each of the last four amino acid residues of PLC*β*3 (^1231^NTQL^1234^-COOH) was mutated to Ala [12]. When treated with ATP for 4 hours, cells transfected with the construct overexpressing wild-type PLC*β*3 (NTQL) showed an increase in IL-1*α* and IL-1ra secretion, whereas the dominant-negative mutant, PLC*β*3 NTQA (L1234A), significantly inhibited their secretion. In contrast, PLC*β*3 ATQL (N1231A), NAQL (T1232A), and NTAL (Q1233A) had no inhibitory effect on IL-1*α* and IL-1ra secretion (Figure 3(b)). These results indicate that the fourth amino acid (Leucine) of the PDZ domain in PLC*β*3 is critical for binding some regulatory protein(s) to control IL-1ra and IL-1*α* secretion and overexpression by the ATP/P2Y_2_ signaling complex. These results show that PLC*β*3 may have a major physiological function in the pathway mediating ATP/P2Y_2_-induced IL-1ra and IL-1*α* secretion, thus, regulating* MUC5AC* gene expression in NCI-H292 cells. P2Y_2_ is a G*α*q-coupled G-protein receptor; therefore, we investigated whether IL-1ra has an effect on P2Y_2_-induced Ca^2+^ influx after treatment with ATP. IL-1ra significantly decreased [Ca^2+^]_i_, but IL-1*α* slightly increased [Ca^2+^]_i_ (Figures 3(a) and 3(b)).

### 3.4. Downstream Signaling by PLC*β*3 of the ERK1/2-CREB Pathway Is Controlled by IL-1ra and IL-1*α*

**Figure 4:**
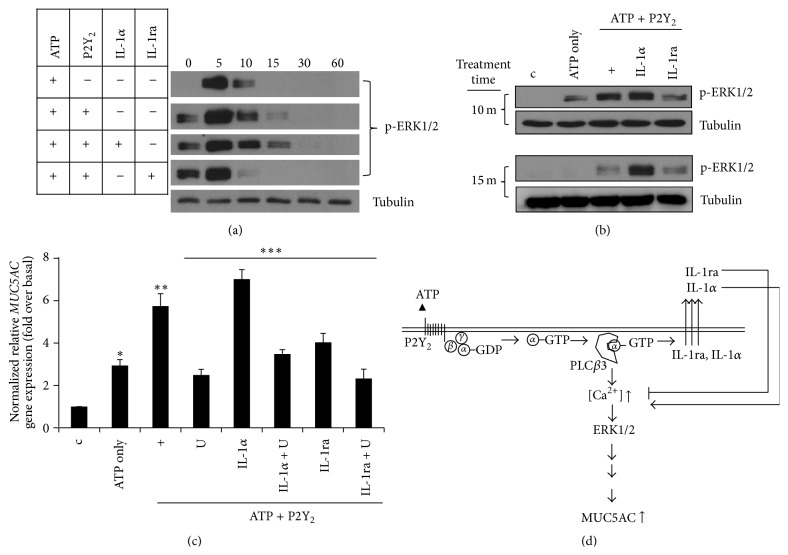
IL-1ra decreases ATP/P2Y_2_-induced* MUC5AC* gene expression by inhibiting ERK1/2 activation. After transfection with wild-type P2Y_2_, the cells were treated with either IL-1ra or IL-1*α* for 4 hours prior to treatment with ATP for different times (a) or either 10 or 15 min (b). ERK1/2 phosphorylation was analyzed by Western blot. Tubulin was used as a loading control. (c) After transfection with wild-type P2Y_2_, the cells were treated with IL-1ra, IL-1*α*, or U0126 (10 *μ*M) for 4 hours prior to treatment with ATP for 24 hours. qRT-PCR for MUC5AC transcript was performed. ^*∗*^ *p* < 0.05 compared to the control, ^*∗∗*^ *p* < 0.05 compared to ATP only, and ^*∗∗∗*^ *p* < 0.05 compared to ATP/P2Y_2_-treated cells. Data shown are representative of three independent experiments. (d) A schematic diagram is presented to show the potential mechanisms for secretion of IL-1ra and IL-1*α*, and their physiological roles during inflammatory responses.

ERK1/2 signaling is critical for ATP/P2Y_2_-induced* MUC5AC* gene expression in airway epithelial cells [12]; therefore, we hypothesized that IL-1ra or IL-1*α* may affect ERK1/2 MAPK activation. Phosphorylation of ERK1/2 was increased by ATP treatment for 5 min but then decreased rapidly. ERK1/2 phosphorylation activated by IL-1*α* was prolonged compared to phosphorylation by the ATP/P2Y_2_ signaling pathway (Figure 4(a)). However, IL-1ra treatment for 10 min inhibited ERK1/2 phosphorylation increased by ATP/P2Y_2_ signaling. Treatment with IL-1*α* or ATP for 10 and 15 min, respectively, had an additive effect on ERK1/2 phosphorylation, which was dramatically inhibited by IL-1ra (Figure 4(b)). Interestingly, treatment with both ATP and IL-1*α* resulted in prolonged activity to maintain ERK1/2 activation, whereas IL-1ra remarkably abolished ATP/P2Y_2_-induced ERK1/2 activation. These results suggest that IL-1*α* prolonged ERK1/2 activation, but IL-1ra had a negative effect on activation. Next, to examine whether ERK1/2 MAPK is essential for IL-1ra- or IL-1*α*-modulated* MUC5AC* gene expression, U0126, a MEK1/2 specific inhibitor, was evaluated (Figure 4(c)). As expected, IL-1ra- and IL-1*α*-mediated gene expression of* MUC5AC* were downregulated by U0126. Thus, ERK1/2 may be associated with IL-1ra- and IL-1*α*-modulated* MUC5AC* gene expression in human airway epithelial cells.

## 4. Discussion

MUC5AC, known as tracheobronchial mucin [21], is a major mucin of lung mucus and is abundantly expressed during acute and chronic lung diseases [22] and thereby greatly contributes to disease morbidity and mortality [6]. As described above, studies regarding MUC5AC have focused on the signaling pathway for gene expression itself by different stimulants, such as proinflammatory cytokines, LPS, endotoxins, cigarette smoking, yellow dust, or airborne particulate matter in several lung cell types. However, there are few reports on regulatory pathways that control MUC5AC secretion or overproduction. An understanding of the regulatory mechanisms that control mucus hypersecretion in respiratory diseases is essential for improving future therapies.

The important proinflammatory role of IL-1 in many human diseases has been described over the past a decade. The balance between IL-1 and IL-1ra has been extensively studied in a variety of experimental animal models of diseases including arthritis, inflammatory bowel disease (IBD), granulomatous and fibrotic lung disorders, infectious diseases, and arterial diseases [4]. Extracellular ATP is known to regulate a number of inter- and intracellular functions in many cells and tissues [23]. ATP is extracellularly secreted in response to LPS-induced airway inflammation in the respiratory system [12]. In several studies, ATP/P2Y_2_ signaling in the airway is a critical sensor for airway exposure to airborne allergens and secretes the IL-33 cytokine into the airway lumen [24]. We hypothesized that ATP/P2Y_2_ signaling may function in maintaining homeostasis by abolishing inflamed microenvironments in the airway or by creating a more severe inflamed condition. Therefore, we tried to identify which proteins or signaling molecules control these processes. We speculated that IL-1ra and IL-1*α*, which have opposite regulatory roles, may also be important in human airway epithelial cells. We found that IL-1ra or IL-1*α* has a negative effect on production and secretion of each other, reciprocally. This is consistent with the results of studies on leukocytes showing that binding of IL-1*α* to IL-1RI results in induction of bioactive IL-1*β* [25, 26] and sIL-1ra [27], whereas sIL-1ra inhibits both the activity and synthesis of IL-1*α* and IL-1*β* in monocytes [28]. These results suggest that overexpression of IL-1*α* and/or underproduction of IL-1ra predisposes one to the development of disease; therefore, the therapeutic administration of IL-1ra is efficacious in preventing tissue damage [4].

P2Y_2_ is a G*α*q-coupled receptor; therefore, an increase in the concentration of Ca^2+^ influx was not surprising result of this study. However, it is notable that secreted IL-1ra or IL-1*α* regulated the concentration of Ca^2+^ influx induced by ATP/P2Y_2_ (Figures 3(a) and 3(b)). More surprisingly, IL-1ra inhibited PLC*β*3 activation (Figure 3(a)). These results suggest that Ca^2+^-PLC*β*3 may be essential for IL-1ra- or IL-1*α*-regulated* MUC5AC* gene expression in airway epithelial cells. Understanding the physiological features of the PDZ domain in PLC*β*3 will provide additional insight into the molecular signaling mechanism that leads to protein complex formation [12]. The PDZ domain is critical for signaling because it depends heavily on the PDZ-binding partner protein(s) to modulate positively/negatively its own signal transduction pathway. Our previous study reported that the PDZ domain in PLC*β*3 is associated with ATP-induced* MUC5AC* gene expression [12]. In the present study, we found that the binding activity of the PDZ domain, and consequently the function of PLC*β*3, is determined by the ability of L1234, leading to secretion of IL-1ra or IL-1*α*, which in turn controls* MUC5AC* gene expression in airway epithelial cells. This is a noteworthy result which suggests that the balance between IL-1ra and IL-1*α* can contribute to an inflamed microenvironment in the airway. For the same reason, PDZ-binding partner protein(s) that can be recruited to increase inflammatory conditions or maintain homeostasis in the respiratory track should further be investigated.

The molecule mediating PLC*β*3 signaling to control IL-1*α*- or IL-1ra-regulated* MUC5AC* gene expression is yet to be elucidated. In our previous studies, ERK1/2 MAPK was identified as a molecule involved in cytokine-induced* mucin* gene expression [12, 13, 29]. Since this protein is tightly regulated by PLC*β*3 in NHNE and NCI-H292 cells [12], its effect on ATP-mediated cytokine signaling also needed to be clarified. The activation of ERK1/2 by IL-1*α* was more prolonged than that of ATP alone (Figures 4(a) and 4(b)), suggesting that ERK1/2 may be essential for ATP/P2Y_2_-induced* MUC5AC* gene expression. This is not surprising since ERK1/2 played an important role as a protein kinase to increase* MUC5AC* gene expression by various cytokines in our system. ATP/P2Y_2_-induced ERK1/2 phosphorylation was inhibited by IL-1ra, resulting in downregulation of ATP/P2Y_2_-induced* MUC5AC* gene expression. Consequently, IL-1ra secretion was affected by PLC*β*3 activation; thus, the secretion of IL-1ra could control* MUC5AC* gene expression by regulating ERK1/2 activity (Figure 4(c)).

Taken together, we suggest that the interaction between ATP and the P2Y_2_ receptor led to the secretion of various cytokines regulating airway inflammation. Not only can ATP induce inflammation, but also it can also amplify and transfer inflammatory signals to nearby epithelial cells in the airway. The G*α*q-PLC*β*3-Ca^2+^ pathway modulated IL-1ra and IL-1*α* secretion to maintain homeostasis. Therefore, the balance between these cytokines up- or downregulated* MUC5AC* gene expression. In this signaling pathway, the PDZ domain in PLC*β*3 may play a crucial role in the development of inflammation in the airway. In addition, secreted IL-1ra diminished IL-1*α* production/secretion and* MUC5AC* gene expression (Figure 4(d)). Thus, the development of a protein or small molecule as a novel therapeutic drug to inhibit ATP/P2Y_2_ signaling could be useful to control airway inflammation.
